# Histidine-rich glycoprotein as an excellent biomarker for sepsis and beyond

**DOI:** 10.1186/s13054-018-2127-5

**Published:** 2018-08-17

**Authors:** Masahiro Nishibori, Hidenori Wake, Hiroshi Morimatsu

**Affiliations:** 1 Department of Pharmacology, Okayama University Graduate School of Medicine, Dentistry and Pharmaceutical Sciences, 2-5-1 Shikata-cho, Kita-ku, Okayama, 700-8558 Japan; 2 Department of Anesthesiology & Resuscitology, Okayama University Graduate School of Medicine, Dentistry and Pharmaceutical Sciences, Okayama, 700-8558 Japan

**Keywords:** Sepsis, Biomarker, Histidine-rich glycoprotein, Diagnosis

## Abstract

Sepsis remains a critical problem with high morbidity and mortality worldwide. One of the problems we have in critical care is the need to find a good biomarker of sepsis to determine the existence of bacterial infection and the severity of patients. This would enable us to start appropriate treatment at an earlier stage of the disease course. We propose that decreases in the plasma protein histidine-rich glycoprotein (HRG) is an excellent biomarker of sepsis compared with the current markers. Based on the novel pathophysiological roles of HRG in the cascade of events during sepsis, we also discuss the potential for supplemental therapy with purified HRG.

## ᅟ

Sepsis remains a critical problem with high morbidity and mortality worldwide [1–3]. As it is a very heterogeneous syndrome, reliable markers are needed, and these could greatly help in the development and evaluation of new treatments [4]. In the case of re-evaluation of activated protein C (APC), the efficacy of the APC against all population of septic patients was denied, but a slight improvement was observed in the subpopulation of patients with highest severity [5].

In critical care we also need to find a good biomarker of sepsis to determine the existence of bacterial infection and the severity of patients [6–8]. This would enable us to start appropriate treatment at an earlier stage of the disease course. Procalcitonin and presepsin are such biomarkers and have been used in clinical practice [1, 6], although plasma presepsin levels may increase if glomerular filtration rate is altered [9].

Recently, we identified a plasma factor, histidine-rich glycoprotein (HRG), as a very important molecule to understand the pathogenesis of sepsis in the cecal ligation and puncture (CLP) mouse model of sepsis [10]. We found that plasma levels of HRG decreased rapidly due to inhibition of transcription of HRG mRNA in the liver, degradation of HRG by thrombin, and HRG deposition on intravascular immunothrombi [10]. HRG plays an important role on neutrophils in maintaining their round shape and smooth cell surface and suppressing spontaneous production of reactive oxygen species. Thus, plasma HRG maintains circulating neutrophils in the quiescent state, easing their passage through capillary vessels and limiting damage to vascular endothelial cells, which may be induced by reactive oxygen species released from neutrophils. However, decreased plasma levels of HRG in sepsis can result in adhesion of neutrophils on vascular endothelial cells, with subsequent intravascular NETosis and immunothrombosis [10]. Moreover, it was demonstrated that HRG not only protected vascular endothelial cells from strong activation and apoptosis induced by lipopolysaccharide or TNF-α [10], but also inhibited Zn^2+^-induced aggregation of erythrocytes [11].

The pathological disorders that occur during sepsis include multiple organ failure associated with coagulopathy and dysregulated inflammation/immune responses [1, 12]. These disorders are likely related to dysregulation of neutrophils, activation and damage of vascular endothelial cells, enhanced platelet aggregation, immune paralysis, and procoagulant activity. It should be mentioned that all these events are mutually related and seem to form a cascade-like reaction. The decrease in plasma HRG appears to be present relatively upstream of these events because supplementary treatment of CLP septic mice with exogenous HRG prevented almost all these responses during septic pathogenesis [10]. Therefore, decreases in plasma levels of HRG may be involved in this cascade of events in polymicrobial infection.

Based on this information, we conducted a clinical study in patients with possible sepsis and showed that plasma HRG levels of the septic patients were significantly lower than in the other patients [13]. Also, plasma HRG levels were superior to those of procalcitonin and presepsin for the estimation of the presence of bacterial infection [13]. Moreover, plasma HRG levels were a good prognostic indicator and were correlated with APACHE II and SOFA scores. Most interestingly, the determination of plasma HRG alone could still predict mortality after adjustment for APACHE II score, and this was not observed with procalcitonin and presepsin [13].

The most important point is that plasma HRG directly relates to the pathogenesis of sepsis, i.e., it has a causal relationship with the development of the cascade of events in sepsis. It is quite possible that we could classify septic patients based on plasma HRG levels, not only to define the severity of patients [13] but also for the entry of patients for evaluating of a new sepsis treatment.

Supplementary therapy with purified HRG was clearly shown to be beneficial in a CLP mouse model [10]. More work should be done before establishing the efficacy of supplementary therapy, but HRG therapy should be relatively safe because HRG itself is an endogenous plasma protein and the treatment aims towards normalization of plasma HRG. The study of plasma HRG will provide novel insights into sepsis pathophysiology.

## Abbreviations

APC: Activated protein C
CLP: Cecal ligation and puncture
HRG: Histidine-rich glycoprotein
